# Overweight and obesity among Brazilian healthcare university students: prevalence and associated factors

**DOI:** 10.20945/2359-3997000000602

**Published:** 2023-02-07

**Authors:** Humberto Carlos de Faria, Anderson Garcez, Juvenal Soares Dias da Costa, Marcelo Ramos, Vera Maria Vieira Paniz

**Affiliations:** 1 Universidade do Vale do Rio dos Sinos Programa de Pós-graduação em Saúde Coletiva São Leopoldo RS Brasil Programa de Pós-graduação em Saúde Coletiva, Universidade do Vale do Rio dos Sinos (Unisinos), São Leopoldo, RS, Brasil; 2 Universidade de Rio Verde Faculdade de Medicina Rio Verde GO Brasil Faculdade de Medicina, Universidade de Rio Verde (UniRV), Rio Verde, GO, Brasil; 3 Universidade Federal de Ciências da Saúde de Porto Alegre Programa de Pós-graduação em Ciências da Nutrição Porto Alegre RS Brasil Programa de Pós-graduação em Ciências da Nutrição, Universidade Federal de Ciências da Saúde de Porto Alegre (UFCSPA), Porto Alegre, RS, Brasil

**Keywords:** Overweight, obesity, prevalence, university students, Brazil

## Abstract

**Objective:**

Obesity is an important factor for cardiovascular and metabolic events. Thus, this study aimed to assess the prevalence of overweight/obesity and the associated factors among healthcare university students.

**Materials and methods:**

A cross-sectional university-based study of 2,245 healthcare university students in the Midwest region of Brazil was conducted in 2018. Overweight and obesity were defined as body mass index (BMI) ≥ 25 kg/m^2^ and BMI ≥ 30 kg/m^2^, respectively. Demographic, socioeconomic, behavioral, family and comorbidities characteristics were investigated. Poisson regression was used for the multivariable analysis. All analysis was stratified by sex.

**Results:**

The mean age of the sample was 22.7 years (standard deviation = 4.1) and 69.5% of the students was female. The prevalence of overweight and obesity were 13.9% (95% confidence interval [CI]: 12.2-15.6) and 4% (95% CI: 3.0-4.9) for women and 34.5% (95% CI: 30.9-38.1) and 11.3% (95% CI: 8.9-13.6) for men, respectively. After multivariate adjustment, the prevalence of overweight/obesity was at least 70% higher in female students aged 24 years or older (prevalence ratio [PR] = 1.73; 95% CI: 1.24-2.41) and those who smoke (PR = 1.95; 95% CI: 1.66-3.02). Additionally, female students with a family history of obesity (PR = 2.01; 95% CI: 1.46-2.77) or with two or three comorbidities (PR = 2.09; 95% CI: 1.43-3.04) exhibited a significantly higher probability for overweight/obesity. Similar findings were observed in male students, but with smaller effect sizes.

**Conclusion:**

This study revealed a high prevalence of overweight/obesity among healthcare university students, especially in men. It was related to the socio-demographic and family characteristics rather than behavioral factors.

## INTRODUCTION

The worldwide marked increase in the prevalence of overweight and obesity in the last decades being an important public health concern (1-5). It has been identified as a modifiable risk factor for cardiovascular diseases and overall mortality (6-9). In addition, the prevalence of overweight and obesity has increased in all age groups, including young adults (10-12).

Overweight and obesity result from an imbalance between energy intake and energy expenditure, consequently leading to weight gain (1,13). Moreover, the etiological mechanisms involved in overweight, and obesity may be related to endocrine factors, as well as environmental, socioeconomic, behavioral, psychosocial, and hereditary aspects (1,4,13).

University students are affected by overweight and obesity (12,14-18). Data from 22 countries in different regions of the world and with different socioeconomic conditions estimated that around 22% of university students have overweight or obesity (15). Overweight and obesity have also been reported among healthcare university students (19-21). However, there is a dearth in the research literature with this specific population group in Brazil. In addition, factors such as being male, advanced age, higher socioeconomic level, inappropriate eating behavior, and low level of physical activity may be potentially associated with overweight and obesity in university students (14-16,22,23).

Thus, the present study aimed to assess the prevalence of overweight and obesity and the associated factors among healthcare university students in the Midwest region of Brazil.

## MATERIALS AND METHODS

This cross-sectional university-based study was conducted in 2018 among healthcare university students. The sample comprised a census of the total population of 2,662 university students of health sciences (medicine, nursing, dentistry, physiotherapy, pharmacy and physical education) from three campuses (Rio Verde, Aparecida de Goiânia e Goianésia) of the University of Rio Verde (UniRV). It is a large private university located in the state of Goiás in the Midwest region of Brazil. The inclusion criteria for participating were university students aged 18 years or older who were enrolled in these six health courses until the second half of 2017. The exclusion criteria were students who were not identified or who refused the invitation to participate in the study. The current study was conducted according to the guidelines laid down in the Declaration of Helsinki, and all procedures involving research study participants were approved by the Research Ethics Committee of the University of Vale do Rio dos Sinos (CAAE: 97545818.2.0000.5344/protocol no. 2.892.764) and the University of Rio Verde (CAAE: 97545818.2.3001.5077/protocol no. 2.905.704). Written informed consent was obtained from all university students.

This study was part of a larger research study entitled “Epidemiological profile of health students of the University of Rio Verde (UniRV), Goiás, 2018”, contemplating a self-administered questionnaire with 202 questions developed by the authors for this specific study, including anthropometric data as well as demographic, socioeconomic, behavioral, family and comorbidities characteristics and others. This questionnaire was self-completed in paper form by all of the students in their classes with a duration of 60 minutes. The data collection was conducted in November 2018 including the support of university professors and with a previous explanation given by investigators who were trained to ensure uniformity and quality in this collection process. If a student was absent from class during the application of the questionnaire, a maximum of three attempts were made to contact and complete questionnaire data.

The international classification of nutritional status was used for the assessment of the outcome-dependent variables. Self-reported anthropometric measures (body weight and height) were used to determine the presence of overweight and obesity. Based on body mass index (BMI; calculated by dividing body weight in kilograms by height in meters squared), overweight and obesity were defined as BMI ≥ 25 kg/m^2^ and BMI ≥ 30 kg/m^2^, respectively (24,25). Independent variables were collected to characterize the sample and to determine the potential factors associated with the outcomes of interest. The demographic variables included sex (male or female), age (collected as a continuous variable and categorized into four age groups), self-reported skin color (classified as white or non-white), and marital status (living with or without a partner). The socioeconomic variables included current occupational status (working or not working) and economic class (categorized using the Economic Classification Criterion of the Brazilian Association of Research Companies-ABEP). This classification criterion estimated the buying power of individuals and families, including the possession of household items and the education level of the householder. The behavioral variables included smoking (smoker, non-smoker and ex-smoker), alcohol consumption (regular or non-regular), physical activity (physically active or sedentary), and consumption of fruits and vegetables (adequate or not adequate). Regarding behavioral variables, we classified current smokers as those smoking at least one cigarette daily. Only those who reported quitting more than one month prior to completing this study were considered ex‐smokers (26). Regular alcohol consumption was determined as at least three times weekly and at least three doses each of alcoholic beverages. We classified physically active as those students who reported at least 150 minutes of moderate-intensity physical activity per week (27). Those who reported the consumption of one or more servings of fruits and vegetables less than three days a week were classified as inadequate (28,29). Family and comorbidities variables included family history of obesity (mother, father or both) and presence of comorbidities (diabetes, hypertension, or dyslipidemia – high cholesterol level). We subsequently categorized the presence of comorbidities as an ordinal (none, one or two/three).

All the data collected were double-entered and validated using version 3.5 of the EpiData software package (EpiData Association, Odense, Denmark). Data analysis was performed using Stata version 12.0 (StataCorp LP, College Station, Texas, USA). Descriptive analysis was used to describe the sample characteristics and prevalence of overweight and obesity. Pearson's chi-square test for heterogeneity of proportions (categorical variable) or linear trend (ordinal variable) was used to explore the potential independent variables associated with overweight and obesity. Further, the classifications of overweight and obesity were grouped as overweight/obesity (BMI ≥ 25 kg/m^2^) in the regression analysis. We used the modified Poisson regression with robust variance to estimate unadjusted and adjusted prevalence ratios with 95% confidence intervals (95% CI). It is an alternative to logistic regression for the analysis of cross-sectional studies with binary outcomes. When working with frequent outcomes, the odds ratio can strongly overestimate the prevalence ratio (30). A conceptual hierarchical model of causality was used to support the entry of variables in the multivariable regression analysis (31). This model considered three levels of determinants for overweight/obesity: demographic and socioeconomic variables were used in the most distal level (first level); behavioral and family variables were considered as intermediate level (second level); whereas presence of comorbidities was included in the proximal level (third level). Each variable was adjusted to the others at the same or previous level in a hierarchical model of causality. Only variables associated with the outcome (overweight/obesity) at P < 0.20 in the unadjusted model were subsequently entered and retained in the final multivariate-adjusted model. All analysis was stratified by sex because of observed significant differences in sample characteristics and prevalence of overweight and obesity between male and female participants. A value of P < 0.05 was considered significant.

## RESULTS

Of the total of 2,662 eligible university students, 356 (13%) were not identified after three attempts or refused to participate. In addition, eleven students (0,4%) were excluded for incorrectly completing the questionnaire and another 50 students (1,9%) because of incomplete anthropometric data. Therefore, a total of 2,245 healthcare university students were included in the final analysis, including 1,561 women (69.5%) and 684 men (30.5%). The mean age of the total sample was 22.7 years (standard deviation = 4.1). Of these, 71% were medical students.

Table 1 shows the general characteristics for the total sample and according to sex. More than 50% of students were less than 22 years old, had white skin color, and did not live with a partner. Most students belong to the economic class A or B and they did not have any kind of employment. Regarding behavioral characteristics, 7.5% were smokers, 16.8% frequently consumed alcohol, and 35% were physically inactive, while 51% and 30.4% had inadequate consumption of fruits and vegetables, respectively. About 30% had at least one family member with obesity, and 24.5% had other comorbidities. Most variables showed a significant difference between male and female university students (Table 1).

**Table 1:** General sample characteristics of healthcare university students in the Midwest of Brazil, 2018 (N = 2,245)

Characteristics		Total (n = 2,245)	Women (n = 1,561)	Men (n = 684)	*P*-value*
		n (%)	n (%)	n (%)	
Age (years)					0.031
	≤20	568 (25.3)	399 (25.6)	169 (24.7)	
	21 a 22	767 (34.2)	556 (35.6)	211 (30.9)	
	23 a 24	498 (22.2)	341 (21.8)	157 (23.0)	
	>24	412 (18.4)	265 (17.0)	147 (21.5)	
Skin color					0.567
	White	1297 (57.8)	908 (58.2)	389 (56.9)	
	Non-white	948 (42.2)	653 (41.8)	295 (43.1)	
Marital status (n = 2,239)					0.486
	Without a partner	1976 (88.3)	1379 (88.6)	597 (87.5)	
	With a partner	263 (11.8)	178 (11.4)	85 (12.5)	
Economic class (ABEP) (n = 2,152)					<0.001
	A (high)	965 (44.8)	617 (41.1)	348 (53.6)	
	B	945 (43.9)	707 (47.0)	238 (36.7)	
	C-E (low)	2442 (11.3)	179 (11.9)	63 (9.7)	
Occupational status (n = 2,209)					0.004
	Not working	1989 (90.0)	1400 (91.3)	589 (87.3)	
	Working	220 (10.0)	134 (8.7)	86 (12.7)	
Smoking (n = 2,195)					<0.001
	Non-smoker	1880 (85.7)	1378 (90.4)	502 (74.9)	
	Ex-smoker	150 (6.8)	82 (5.4)	68 (10.2)	
	Smoker	165 (7.5)	65 (4.3)	100 (14.9)	
Alcohol consumption (n = 2,238)					<0.001
	Non-regular	1863 (83.2)	1347 (86.5)	516 (75.9)	
	Regular	375 (16.8)	211 (13.5)	164 (24.1)	
Physical activity (n = 2,173)					<0.001
	Physically active	1413 (65.0)	922 (61.2)	491 (73.7)	
	Sedentary	760 (35.0)	585 (38.8)	175 (26.3)	
Consumption of fruits					<0.001
	Adequate	1089 (48.5)	812 (52.0)	277 (40.5)	
	Not adequate	1156 (51.5)	749 (48.0)	407 (59.5)	
Consumption of vegetables					0.264
	Adequate	1563 (69.6)	1098 (70.3)	465 (68.0)	
	Not adequate	682 (30.4)	463 (29.7)	219 (32.0)	
Family history of obesity (n = 2,218)					0.371
	No	1564 (70.5)	1104 (71.4)	460 (68.5)	
	Mother or father	489 (22.1)	331 (21.4)	158 (23.5)	
	Mother and father (both)	165 (7.4)	111 (7.2)	54 (8.0)	
Hypertension (n = 2,244)					<0.001
	No	2152 (95.9)	1523 (97.6)	629 (92.1)	
	Yes	92 (4.1)	38 (2.4)	54 (7.9)	
Dyslipidemia (cholesterol) (n = 2,244)					0.010
	No	1803 (80.4)	1232 (78.9)	571 (83.6)	
	Yes	441 (19.7)	329 (21.1)	112 (16.4)	
Diabetes (n = 2,244)					0.389
	No	2132 (95.0)	1479 (94.8)	653 (95.6)	
	Yes	112 (5.0)	82 (5.2)	30 (4.4)	
Presence of comorbidities (n = 2,244)					0.533
	No	1693 (75.5)	1173 (75.1)	520 (76.1)	
	One	463 (20.6)	330 (21.1)	133 (19.5)	
	Two/three	88 (3.9)	58 (3.7)	30 (4.4)	
Nutritional status (BMI)					<0.001
	Normal (<25 kg/m^2^)	1653 (73.6)	1282 (82.1)	371 (54.2)	
	Overweight (25 to 29,9 kg/m^2^)	453 (20.2)	217 (13.9)	236 (34.5)	
	Obesity (≥30 kg/m^2^)	139 (6.2)	62 (4.0)	77 (11.3)	

ABEP: Economic Classification Criterion of the Brazilian Association of Research Companies; BMI: body mass index.

Presence of comorbidities = Hypertension + Dyslipidemia + Diabetes

* *P*-values for chi-square test for heterogeneity of proportions.

NOTE: Differences in variable sample sizes (n) due to missing data.

The overall prevalence rate of overweight and obesity for this sample was 20.2% (95% CI: 18.5-21.8) and 6.2% (95% CI: 5.2-7.2), respectively. The frequency of this condition was higher in male than in female students. The prevalence of overweight and obesity were 13.9% (95% CI: 12.2-15.6) and 4% (95% CI: 3.0-4.9) for women and 34.5% (95% CI: 30.9-38.1) and 11.3% (95% CI: 8.9-13.6) for men, respectively. Table 2 shows the proportions of nutritional status according to the demographic, socioeconomic, behavioral, family and comorbidities characteristics for men and women separately. In both sexes, a significant and direct association was observed between overweight and obesity with age, family history of obesity and the presence of comorbidities: a higher prevalence were observed in older students (aged > 24 years), students with mother and father with history of obesity and among those with two or three comorbidities. Marital status also showed a significant association with overweight and obesity in both sexes; a higher prevalence was observed in students who reported living with a partner. Overweight and obesity were also significantly associated with economic class, smoking behavior and physical activity in female students only: a higher prevalence of overweight and obesity was observed in female students with low economic class (inverse association) as well as a higher prevalence of overweight was observed in female ex-smokers and those classified as physically active and a higher prevalence of obesity in female smokers and those classified as sedentary (Table 2).

**Table 2:** Prevalence of overweight (BMI ≥ 25 kg/m^2^) and obesity (BMI ≥ 30 kg/m^2^), stratified by sex, according to sample characteristics of healthcare university students in the Midwest region of Brazil, 2018 (N = 2,245)

Characteristics		Women (n = 1,561)				Men (n = 684)			
		Nutritional status				Nutritional status			
		Normal n (%)	Overweight n (%)	Obesity n (%)	*P*-value*	Normal n (%)	Overweight n (%)	Obesity n (%)	*P*-value*
Age (years)					<0.001				<0.001
	≤20	342 (85.7)	45 (11.3)	12 (3.0)		107 (63.3)	51 (30.2)	11 (6.5)	
	21 a 22	465 (83.6)	76 (13.7)	15 (2.7)		122 (57.8)	70 (33.2)	19 (9.0)	
	23 a 24	277 (81.2)	47 (13.8)	17 (5.0)		78 (49.7)	61 (38.9)	18 (11.5)	
	>24	198 (74.7)	49 (18.5)	18 (6.8)		64 (43.5)	54 (36.7)	29 (19.7)	
Skin color					0.574				0.410
	White	741 (81.6)	127 (14.0)	40 (4.4)		218 (56.0)	126 (32.4)	45 (11.6)	
	Non-white	541 (82.9)	90 (13.8)	22 (3.4)		153 (51.9)	110 (37.3)	32 (10.9)	
Marital status					0.037				0.001
	Without a partner	1145 (83.0)	181 (13.1)	53 (3.8)		339 (56.8)	196 (32.8)	62 (10.4)	
	With a partner	134 (75.3)	35 (19.7)	9 (5.1)		30 (35.3)	40 (47.1)	15 (17.7)	
Economic class (ABEP)					0.036				0.350
	A (high)	514 (83.3)	76 (12.3)	27 (4.4)		190 (54.6)	119 (34.2)	39 (11.2)	
	B	587 (83.0)	98 (13.9)	22 (3.1)		134 (56.3)	79 (33.2)	25 (10.5)	
	C-E (low)	133 (74.3)	35 (19.6)	11 (6.2)		27 (42.9)	28 (44.4)	8 (12.7)	
Occupational status					0.058				0.982
	Not working	1160 (82.9)	187 (13.4)	53 (3.8)		321 (54.5)	203 (34.5)	65 (11.0)	
	Working	100 (74.6)	26 (19.4)	8 (6.0)		47 (54.7)	29 (33.7)	10 (11.6)	
Smoking					0.003				0.070
	Non-smoker	1147 (83.2)	182 (13.2)	49 (3.6)		284 (56.6)	162 (32.3)	56 (11.2)	
	Ex-smoker	57 (69.5)	19 (23.2)	6 (7.3)		38 (55.9)	22 (32.4)	8 (11.8)	
	Smoker	47 (72.3)	12 (18.5)	6 (9.2)		42 (42.0)	47 (47.0)	11 (11.0)	
Alcohol consumption					0.253				0.215
	Non-regular	1098 (81.5)	192 (14.3)	57 (4.2)		289 (56.0)	171 (33.1)	56 (10.9)	
	Regular	181 (85.8)	25 (11.9)	5 (2.4)		79 (48.2)	64 (39.0)	21 (12.8)	
Physical activity					0.001				0.111
	Physically active	758 (82.2)	139 (15.1)	25 (2.7)		273 (55.6)	173 (35.2)	45 (9.2)	
	Sedentary	485 (82.9)	64 (10.9)	36 (6.2)		92 (52.6)	57 (32.6)	26 (14.9)	
Consumption of fruits					0.650				0.279
	Adequate	667 (82.1)	116 (14.3)	29 (3.6)		160 (57.8)	90 (32.5)	27 (9.8)	
	Not adequate	615 (82.1)	101 (13.5)	33 (4.4)		211 (51.8)	146 (35.9)	50 (12.3)	
Consumption of vegetables					0.858				0.080
	Adequate	899 (81.9)	156 (14.2)	43 (3.9)		250 (53.8)	170 (36.6)	45 (9.7)	
	Not adequate	383 (82.7)	61 (13.2)	19 (4.1)		121 (55.3)	66 (30.1)	32 (14.6)	
Family history of obesity					<0.001				<0.001
	No	952 (86.2)	124 (11.2)	28 (2.5)		281 (61.1)	149 (32.4)	30 (6.5)	
	Mother or father	244 (73.7)	60 (18.1)	27 (8.2)		64 (40.5)	63 (39.9)	31 (19.6)	
	Mother and father (both)	76 (68.5)	28 (25.2)	7 (6.3)		20 (37.0)	19 (35.2)	15 (27.8)	
Presence of comorbidities					<0.001				<0.001
	No	994 (84.7)	147 (12.5)	32 (2.7)		312 (60.0)	170 (32.7)	38 (7.3)	
	One	249 (75.4)	57 (17.3)	24 (7.3)		53 (39.9)	53 (39.9)	27 (20.3)	
	Two/three	39 (67.2)	13 (22.4)	6 (10.3)		6 (20.0)	12 (40.0)	12 (40.0)	

ABEP: Economic Classification Criterion of the Brazilian Association of Research Companies; BMI: body mass index.

Presence of comorbidities = Hypertension + Dyslipidemia + Diabetes.

* *P*-values for chi-square test for heterogeneity of proportions (categorical variables) and for linear trend (ordinal variables).

Table 3 shows the unadjusted and adjusted prevalence ratios estimates for the association between overweight/obesity and the independent variables investigated. After multivariate adjustment, the prevalence of overweight/obesity showed a direct association with age, smoking behavior, family history of obesity, and presence of comorbidities in both sexes. Overweight/obesity was at least 70% higher in female students aged 24 years or older (PR = 1.73; 95% CI: 1.24-2.41) and who smoke (PR = 1.95; 95% CI: 1.66-3.02). Additionally, female students with a family history of obesity (PR = 2.01; 95% CI: 1.46-2.77) or with two or three comorbidities (PR = 2.09; 95% CI: 1.43-3.04) exhibited a significantly higher probability for overweight/obesity. Similar findings were observed in male students, but with smaller effect sizes. Furthermore, male students who reported living with a partner had a 35% higher probability of overweight/obesity (PR = 1.35; 95% CI: 1.11-1.64) than those living without a partner (Table 3).

**Table 3:** Unadjusted and adjusted values of prevalence ratios (PR) with 95% confidence intervals (95% CI) for overweight/obesity (BMI ≥ 25 kg/m^2^), stratified by sex, according to sample characteristics of healthcare university students in the Midwest region of Brazil, 2018 (N = 2,245)

Characteristics		Women (n = 1,561)			Men (n = 684)		
		%	Unadjusted PR (95% CI)	Adjusted* PR (95% CI)	%	Unadjusted PR (95% CI)	Adjusted* PR (95% CI)
**Level 1**							
Age (years)			*P* < 0.001	*P* = 0.001		*P* < 0.001	*P* < 0.001
	≤20	14.3	1 (ref.)	1 (ref.)	36.7	1 (ref.)	1 (ref.)
	21 a 22	16.4	1.15 (0.85, 1.55)	1.22 (0.89, 1.66)	42.2	1.15 (0.89, 1.48)	1.22 (0.94, 1.58)
	23 a 24	18.8	1.31 (0.95, 1.82)	1.33 (0.94, 1.87)	50.3	1.37 (1.07, 1.76)	1.46 (1.13, 1.88)
	>24	25.3	1.77 (1.29, 2.43)	1.73 (1.24, 2.41)	56.5	1.54 (1.21, 1.96)	1.55 (1.20, 2.00)
Skin color			*P* = 0.528			*P* = 0.278	*P* = 0.254
	White	18.4	1 (ref.)	--	44.0	1 (ref.)	1 (ref.)
	Non-white	17.2	0.93 (0.75, 1.16)		48.1	1.10 (0.93, 1.29)	1.10 (0.93, 1.30)
Marital status			*P* = 0.011	*P* = 0.074		*P* < 0.001	*P* = 0.003
	Without a partner	17.0	1 (ref.)	1 (ref.)	43.2	1 (ref.)	1 (ref.)
	With a partner	24.7	1.46 (1.10, 1.93)	1.30 (0.98, 1.72)	64.7	1.50 (1.25, 1.80)	1.35 (1.11, 1.64)
Economic class (ABEP)			*P* = 0.031	*P* = 0.177		*P* = 0.293	*P* = 0.420
	A (high)	16.7	1 (ref.)	1 (ref.)	45.4	1 (ref.)	1 (ref.)
	B	17.0	1.02 (0.80, 1.29)	0.99 (0.77, 1.26)	43.7	0.96 (0.80, 1.16)	0.94 (0.78, 1.12)
	C-E (low)	25.7	1.54 (1.13, 2.09)	1.36 (0.99, 1.87)	57.1	1.26 (0.99, 1.60)	1.25 (0.98, 1.60)
Occupational status			*P* = 0.017	*P* = 0.091		*P* = 0.979	
	Not working	17.1	1 (ref.)	1 (ref.)	45.5	1 (ref.)	--
	Working	25.4	1.48 (1.08, 2.02)	1.32 (0.96, 1.82)	45.4	1.00 (0.78, 1.28)	
**Level 2**							
Smoking			*P* = 0.001	*P* < 0.001		*P* = 0.027	*P* = 0.016
	Non-smoker	16.8	1 (ref.)	1 (ref.)	43.4	1 (ref.)	1 (ref.)
	Ex-smoker	30.5	1.82 (1.28, 2.57)	1.79 (1.25, 2.55)	44.1	1.02 (0.76, 1.35)	1.04 (0.79, 1.37)
	Smoker	27.7	1.65 (1.10, 2.49)	1.95 (1.26, 3.02)	58.0	1.34 (1.10, 1.62)	1.30 (1.06, 1.58)
Alcohol consumption			*P* = 0.133	*P* = 0.103		*P* =0.079	*P* = 0.152
	Non-regular	18.5	1 (ref.)	1 (ref.)	44.0	1 (ref.)	1 (ref.)
	Regular	14.2	0.77 (0.54, 1.09)	0.73 (0.50, 1.07)	51.8	1.18 (0.99, 1.41)	1.14 (0.95, 1.37)
Physical activity			*P* = 0.735			*P* =0.852	
	Physically active	17.9	1 (ref.)	--	47.9	1 (ref.)	--
	Sedentary	17.7	0.99 (0.73, 1.33)		41.7	0.87 (0.72, 1.06)	
Consumption of fruits			*P* = 0.986			*P* =0.127	*P* = 0.203
	Adequate	17.9	1 (ref.)	--	42.2	1 (ref.)	1 (ref.)
	Not adequate	17.9	1.00 (0.81, 1.24)		48.2	1.14 (0.96, 1.35)	1.11 (0.94, 1.32)
Consumption of vegetables			*P* = 0.691			*P* = 0.716	
	Adequate	18.1	1 (ref.)	--	46.2	1 (ref.)	--
	Not adequate	17.3	0.95 (0.75, 1.21)		44.8	0.97 (0.81, 1.16)	
Family history of obesity			*P* < 0.001	*P* < 0.001		*P* < 0.001	*P* < 0.001
	No	13.8	1 (ref.)	1 (ref.)	38.9	1 (ref.)	1 (ref.)
	Mother or father	26.3	1.90 (1.51, 2.41)	1.77 (1.39, 2.27)	59.5	1.53 (1.29, 1.82)	1.43 (1.20, 1.70)
	Mother and father (both)	31.5	2.29 (1.68, 3.13)	2.01 (1.46, 2.77)	63.0	1.62 (1.28, 2.05)	1.63 (1.29, 2.07)
**Level 3**							
Presence of comorbidities			*P* < 0.001	*P* < 0.001		*P* < 0.001	*P* < 0.001
	No	15.3	1 (ref.)	1 (ref.)	40.0	1 (ref.)	1 (ref.)
	One	24.6	1.61 (1.27, 2.03)	1.59 (1.25, 2.03)	60.2	1.50 (1.26, 1.79)	1.38 (1.16, 1.64)
	Two/three	32.8	2.15 (1.45, 3.18)	2.09 (1.43, 3.04)	80.0	2.00 (1.62, 2.46)	1.66 (1.31, 2.09)

ABEP: Economic Classification Criterion of the Brazilian Association of Research Companies; BMI: body mass index.

Presence of comorbidities = Hypertension + Dyslipidemia + Diabetes.

*P*-values for Wald test for heterogeneity of proportions (categorical variables) and for linear trend (ordinal variables) obtained using Poisson regression with robust variance.

* Each variable was adjusted to the others at the same or previous level in a hierarchical model of causality. Only variables associated with the overweight/obesity at *P* < 0.20 in the unadjusted model were subsequently entered and retained in the final multivariate-adjusted model.

In additional analyses (not included), we observed that results were of the same direction and greater magnitude when restricting analyses to only obesity events as the outcome (BMI ≥ 30 kg/m^2^). However, some associations could not be identified due to lack of power caused by a low number of obesity cases in the sample.

## DISCUSSION

In this study, we revealed a high prevalence of overweight/obesity among healthcare university students in the Midwest region of Brazil. We found that the frequency of this condition was higher in male than in female students and that it was related to sociodemographic and family characteristics rather than behavioral factors. Overweight and obesity were associated with age, smoking behavior, family history of obesity and presence of comorbidities in both sexes.

A prevalence of 20.2% and 6.2% for overweight and obesity was found among healthcare university students in this study, respectively. Findings in the same direction were obtained by previous studies conducted among Brazilian medical students from private universities (19,20). Cafure and cols. identified a prevalence of 26.4% and 7.8% for overweight and obesity in medical students from a university of Campo Grande/MS, respectively (20), while another study showed a prevalence of 16.4% and 1.4% for overweight and obesity in medical students from a university of Vitoria-ES, respectively (19). Similar findings were also observed in studies conducted among university students from non-health courses (14-16). Sira and Pawlak identified that 21.3% and 10.8% of students from the University of North Carolina have overweight and obesity, respectively (14), while a prevalence of 26.8% and 10.7% for overweight and obesity, respectively, was observed in a sample of university students in India (16). In addition, around 22% of a sample of university students from 22 countries have overweight or obesity (15).

The present study showed that the prevalence of overweight and obesity was significantly higher among male students (34.5% and 11.3%, respectively), than among female students (13.9% and 4%, respectively). Similarly, previous studies have also found that the prevalence of overweight and obesity tends to be higher among men than among women (14,15,17,18). Peltzer and cols. revealed that the prevalence of overweight and obesity was 18.9% and 5.8% among male students, respectively, while that among female students was 14.1% and 5.2%, respectively (15). The higher prevalence of overweight/obesity in men may be partially explain by the fact that male students are usually satisfied with their weight and body image, considering that they want to increase muscle and weight gain whereas female students are more likely to report attempting to lose weight (32,33).

Our multivariate analysis demonstrated that overweight and obesity were associated with age, smoking behavior, family history of obesity and presence of comorbidities in both sexes. In particular, overweight and obesity were associated with marital status in male students only. Although this study included a sample of young students, we found an association between increased age and the occurrence of overweight and obesity, especially among individuals aged 24 years or older. This relationship has been reported in previous studies (34,35). This is important because the prevalence of overweight and obesity has increased among young adults in recent decades (10-12,36). Furthermore, there is evidence that the transition between adolescence and early adulthood represents a high-risk period for the onset of obesity, regardless of characteristics such as sex and ethnicity (11,36).

Male students who reported living with a partner had a 35% higher probability of overweight/obesity in this study. This finding corroborates previous evidence that marital status is an important factor for overweight/obesity among university students (22,35). Married men were more likely to have overweight and obesity than individuals who were unmarried (37,38). This may be related to behavioral changes when people get married. However, this relationship is complex and cannot be attributed to a single reason.

Regarding behavioral factors, smoking stands out as an important factor associated with the occurrence of overweight and obesity. A positive association between smoking and increased BMI has been reported, especially among university women (39,40). Thus, smoking has been identified as an important risk factor or predictor for the development of obesity in adolescents and young adults (41,42). The active and passive smoking has been associated with increased inflammation, oxidative stress, platelet and endothelial dysfunction, potentially triggering changes in mechanisms involved in the development of cardiovascular risk factors, such as obesity, diabetes and atherosclerosis (41,43). We found a higher prevalence of smoking among male students as well as overweight and obesity. However, a stronger association between smoking and overweight or obesity was found among female students. Based on this finding, we hypothesized that female students with overweight and obesity smoke to lose weight or control weight gain. However, this interpretation must be considered with caution, given the cross-sectional nature and the relatively small sample size of males involved in this study.

This study demonstrated that the occurrence of overweight and obesity tends to be significantly higher among students in which both parents have obesity. Previous studies have shown similar results, with a higher obesity risk among students with a family history of obesity (20,21). This finding demonstrates the important role that heredity plays in the increased likelihood of cardiovascular problems, including increased BMI and obesity (44). Despite family history being a non-modifiable risk factor for obesity, parents are essentially responsible for the development of health behaviors and habits aimed at preventing obesity (45). As such, we consider this aspect as a proxy indicator for family nutrition behaviors: more than 45% of the students lived with their parents or family. Therefore, this aspect was included in the second level of the hierarchical model of causality for overweight and obesity.

For the associations between the comorbidities and the occurrence of overweight and obesity, an increase was found among students who were overweight and obesity, including hypertension, high cholesterol and diabetes. Similar results were observed in a study with medical students (19). Our findings reinforced that the presence of obesity is linked to other comorbidities such as diabetes, hypertension and coronary artery disease, even among young adults (46). In addition, the concomitant presence of cardiovascular risk factors is reported among individuals with obesity, and these risks increases metabolic dysfunction as the degree of adiposity increases (47,48).

We found similar associations in both male and female students for factors associated with overweight and obesity. However, the magnitudes of the associations were smaller among male students. Thus, in addition to the findings of a longitudinal study (11), we hypothesized that adolescence obesity may persist into adulthood, despite the rapid linear growth in males after puberty and the profound changes in women during this period of life. Although this hypothesis is plausible, it requires further in-depth investigation. In addition, differences between the sexes in this study may be attributed to a small sample size of male students.

To our knowledge, this study is one of the first to assess the prevalence of overweight/obesity and the associated factors among healthcare students from a private university located in the state of Goiás in the Midwest region of Brazil. The strengths of this study were the large representative sample of Brazilian healthcare students. In contrast, most of the previous studies were conducted with smaller sample sizes. We used a standardized instrument to obtain the necessary information through a self-administered questionnaire, including demographic, socioeconomic, behavioral, family and comorbidities characteristics. In addition, an appropriated multivariable analysis was used to determine the potential factors associated with overweight and obesity. However, some limitations need to be highlighted. The cross-sectional design limited our ability to make causal inferences. Thus, a reverse causation cannot be ruled out. Therefore, all associations found in this study should be treated with caution. Future longitudinal investigations may help establish the relationships investigated. Self-reported anthropometric measures were used to define the presence of overweight and obesity, which may be considered an important limitation. However, previous studies indicated validity of this information in comparison to the measured data (49,50). Finally, the external validity of this study is limited; it was conducted with a sample of healthcare university students, which does not represent the general population of university students. In addition, the sample of this study consisted mainly of medical students (71%), considering that medical students have reported the lack of time as a major barrier for health habits and quality of life due to the greater demands imposed by the medical course (51,52). Despite these limitations, the present study contributed to scientific knowledge regarding the prevalence of overweight and obesity among healthcare university students. Future confirmatory studies are needed to ratify our findings and to support preventive actions for this specific population group.

In conclusion, this study demonstrated a high prevalence of overweight and obesity among healthcare university students in the Midwest region of Brazil. We found that the frequency of overweight and obesity was higher in male than in female students, and it was related to sociodemographic and family characteristics rather than behavioral factors. Overweight and obesity were associated with age, smoking behavior, family history of obesity, and presence of comorbidities in both sexes.
